# CHANGING FAMILY DESTINIES, DIVERGENT FAMILY CAREGIVING PATTERNS: DO BIRTH COHORTS, GENDER, RACE, AND SES MATTER?

**DOI:** 10.1093/geroni/igac059.2556

**Published:** 2022-12-20

**Authors:** Haoshu Duan

**Affiliations:** University of Maryland, Baltimore, Maryland, United States

## Abstract

Over the past few decades, Americans have experienced a series of demographic transitions include prolonged longevity and rise in the complexities of family structures. The Baby Boomer cohort is at the forefront of these transitions, which has profound implications on their later-life family relations and practices of family caregiving. Most caregiving literature has focused on static care experiences over a short time, while neglecting long-term care experiences. Using 10 waves of longitudinal data from HRS (2000-2018) and latent profile analysis, I identified five prominent long-term caregiving patterns: light parental caregivers (44.1%), intensive spousal caregivers (5.6%), sandwiched caregivers (5.5%), light grandchildren caregivers (38.7%), and heavy grandchildren caregivers (6.2%). Further, I conduct multinominal logistic regression to investigate how birth cohorts, gender, race, and education shaped these patterns. Results suggested that later cohorts have seen a decline in intensive spousal caregivers, light and heavy grandchildren caregivers, but an increase in light parental caregivers. Women are more likely to be sandwiched caregivers than men, and black caregivers are more likely to be intensive spousal caregivers, heavy grandchildren caregivers, and sandwiched caregivers than white. By contrast, white and more educated caregivers are more likely to be light parental caregivers, and this pattern becomes more pronounced in later cohorts. The findings suggest divergent destinies of family caregiving patterns among later cohorts. More disadvantaged groups are shouldering heavier care responsibilities than advantaged groups. Targeted care services should be implemented to ease the care burdens of the vulnerable population.

